# Pharmacodynamics of Ceftibuten: An Assessment of an Oral Cephalosporin against Enterobacterales in a Neutropenic Murine Thigh Model

**DOI:** 10.3390/antibiotics10020201

**Published:** 2021-02-19

**Authors:** Maxwell J. Lasko, Tomefa E. Asempa, David P. Nicolau

**Affiliations:** 1 Center for Anti-Infective Research and Development, Hartford Hospital, 80 Seymour Street, Hartford, CT 06102, USA; maxwell.lasko@hhchealth.org (M.J.L.); tomefa.asempa@hhchealth.org (T.E.A.); 2 Division of Infectious Diseases, Hartford Hospital, Hartford, CT 06102, USA

**Keywords:** pharmacokinetics, pharmacodynamics, Gram-negative, beta-lactamase

## Abstract

Efforts to develop and pair novel oral β-lactamase inhibitors with existing β-lactam agents to treat extended spectrum β-lactamase (ESBL) and carbapenemase-producing Enterobacterales are gaining ground. Ceftibuten is an oral third-generation cephalosporin capable of achieving high urine concentrations; however, there are no robust data describing its pharmacodynamic profile. This study characterizes ceftibuten pharmacokinetics and pharmacodynamics in a neutropenic murine thigh infection model. Enterobacterales isolates expressing no known clinically-relevant enzymatic resistance (*n* = 7) or harboring an ESBL (*n* = 2) were evaluated. The ceftibuten minimum inhibitory concentrations (MICs) were 0.03–4 mg/L. Nine ceftibuten regimens, including a human-simulated regimen (HSR) equivalent to clinical ceftibuten doses of 300 mg taken orally every 8 h, were utilized to achieve various *f*T > MICs. A sigmoidal E_max_ model was fitted to *f*T > MIC vs. change in log_10_ CFU/thigh to determine the requirements for net stasis and 1-log_10_ CFU/thigh bacterial burden reduction. The growth of the 0 h and 24 h control groups was 5.97 ± 0.37 and 8.51 ± 0.84 log_10_ CFU/thigh, respectively. Ceftibuten HSR resulted in a -0.49 to -1.43 log_10_ CFU/thigh bacterial burden reduction at 24 h across the isolates. Stasis and 1-log_10_ CFU/thigh reduction were achieved with a *f*T > MIC of 39% and 67%**,** respectively. The *f*T > MIC targets identified can be used to guide ceftibuten dosage selection to optimize the likelihood of clinical efficacy.

## 1. Introduction

Gram-negative Enterobacterales that harbor extended spectrum β-lactamases (ESBLs) and carbapenemases continue to be a burden on healthcare [1,2,3]. ESBLs, in particular, are frequent causes of infection in both hospitalized and community-dwelling patients [1,3]. Few oral therapeutic options exist, resulting in the use of intravenous broad-spectrum antibiotics (i.e., carbapenems) with the potential for further resistance development [4,5]. Moreover, infections caused by ESBL-harboring Enterobacterales are associated with an approximately two-fold increase in the cost of hospitalization and the length of stay compared with β-lactamase naïve infections [6].

The clear need for carbapenem-sparing antibiotics that can effectively treat ESBL-harboring Enterobacterales has spurred numerous oral drug development efforts over the past several years [7]. Additionally, the availability of an effective oral agent targeting these challenging organisms would provide a significant therapeutic breakthrough for patients treated outside the institutional setting. Thus, repurposing older and infrequently utilized oral β-lactam agents, such as ceftibuten, cefixime, and cefpodoxime, to be paired with investigational β-lactamase inhibitors (BLIs), has become an attractive option.

Ceftibuten has been explored as a β-lactam (BL) backbone because of its excellent bioavailability (75–90%) and high fractional excretion in urine [8,9]. In addition, ceftibuten demonstrates improved in vitro stability against ESBLs compared with other oral third-generation cephalosporins [8,9]. Ceftibuten’s pharmacodynamic driver is free drug time above MIC (*f*T > MIC). The *f*T > MIC requirements that best correlate with cephalosporin efficacy vary from 40–50%, as reported by the European Committee on Antimicrobial Susceptibility Testing (EUCAST) scientific committee [10], to as high as 50–70% [11,12]. However, a formal ceftibuten pharmacodynamic assessment against clinically-relevant Enterobacterales has never been performed. Previously published studies have focused on the activity of ceftibuten in combination with BLIs, with a focus on understanding BLI exposure requirements for efficacy [13,14]. Thus, ceftibuten-specific pharmacodynamic profiling is needed in order to better understand the scope and potential utility of a range of exposures prior to their combination with a novel BLI. Herein, we describe a pharmacodynamic assessment of ceftibuten in a neutropenic murine thigh infection model.

## 2. Results

### 2.1. Murine Pharmacokinetic Studies 

**Figure 1:**
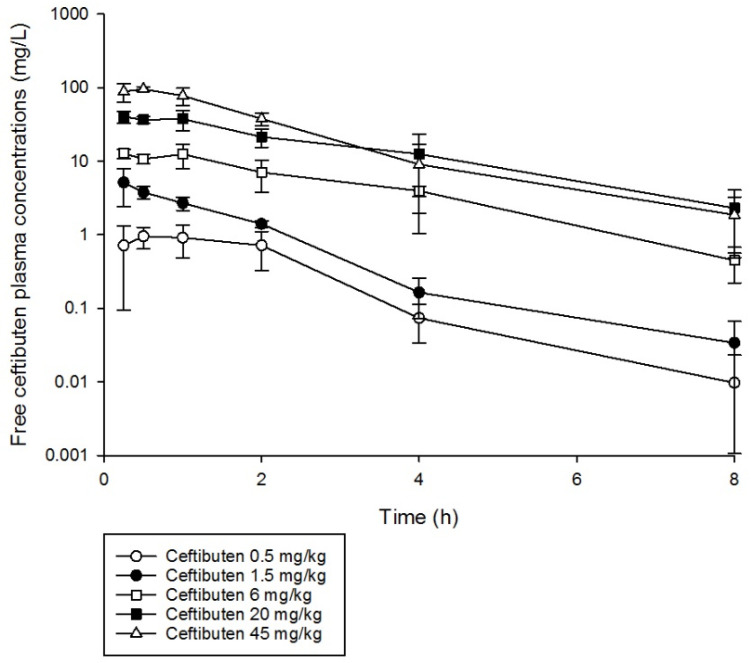
Ceftibuten free plasma concentration–time profiles in a neutropenic mouse thigh infection model following the administration of single subcutaneous doses (0.5–45 mg/kg).

Ceftibuten single doses were well characterized using a uniform one-compartment model with first-order absorption and elimination (Figure 1). Furthermore, the pharmacokinetics of ceftibuten were relatively linear (area under the curve (AUC) R^2^ 0.98) over the single doses administered using non-compartmental analysis (Table 1). The elimination half-life ranged from 1.0 to 1.8 h. The mean (± standard deviation (SD)) pharmacokinetic parameters, namely, volume of distribution (V_d_), 0.342 ± 0.09 (L/kg); absorption constant (K_a_), 5.45 ± 2.46 (1/h); and elimination constant (K_e_), 0.60 ± 0.17 (1/h), were used to simulate ceftibuten regimens and obtain concentration–time profiles. Comparisons of the %*f*T > MIC values achieved with ceftibuten at MICs ranging between 0.03 and 4 mg/L, as well as the area under the concentration–time curve from 0 to 24 h for the free, unbound fraction of the drug (*f*AUC_0–24_), in mice receiving the selected regimens are presented in Table 2.

### 2.2. Pharmacodynamic Studies

**Figure 2:**
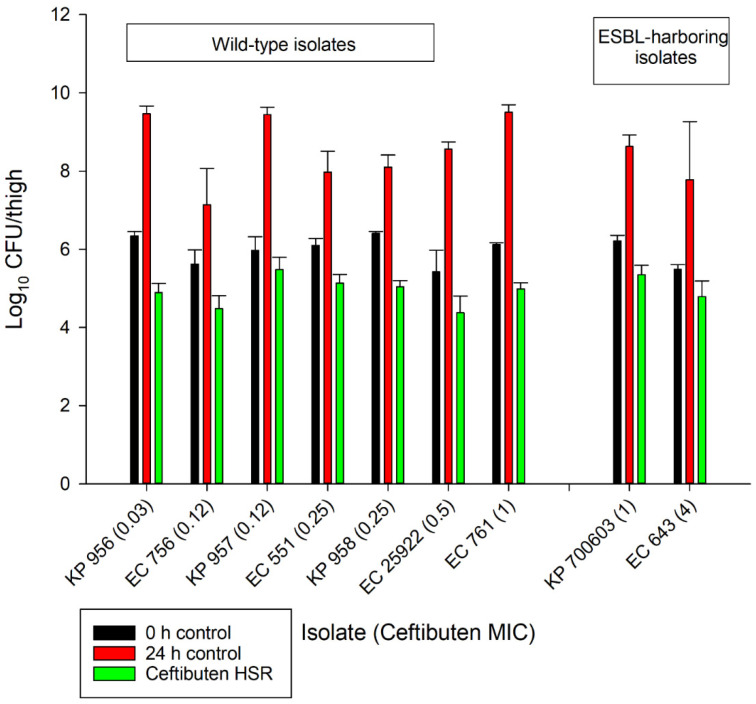
In vivo antibacterial activity of the ceftibuten human-simulated regimen (300 mg taken orally every 8 h) compared to the 0 h and 24 h control groups.

**Figure 3:**
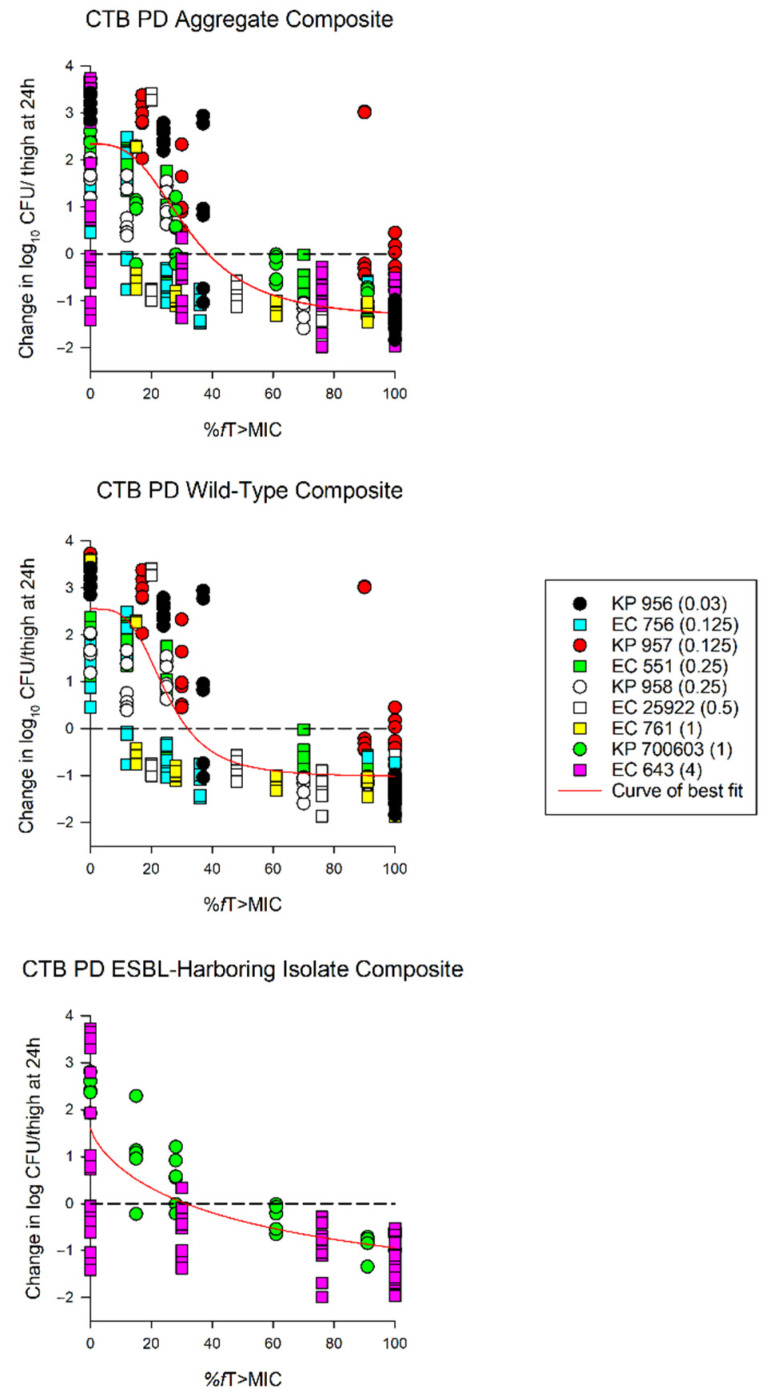
Sigmoid E_max_ curves depicting %*f*T > MIC and change in log_10_ CFU/thigh for all isolates (aggregate composite), wild-type isolates, and extended spectrum β-lactamase (ESBL)-harboring isolates. Each dot represents the log_10_ CFU/thigh for each bacterial strain (ceftibuten MIC) per regimen. KP—*Klebsiella pneumoniae*; EC—*Escherichia coli* (EC). Aggregate composite: R^2^ = 0.79, EC_50_ = 32.46%, and E_max_ = 2.34 log_10_ CFU/thigh; wild-type composite: R^2^ = 0.83, EC_50_ = 24.92%, and E_max_ = 2.55 log_10_ CFU/thigh; ESBL composite: R^2^ = 0.64, EC_50_ = 58.87%, and E_max_ = 2.34 log_10_ CFU/thigh.

The average growth (± standard deviation) of the 0 h and 24 h control groups were 5.97 ± 0.37 log_10_ CFU/thigh and 8.51 ± 0.84 log_10_ CFU/thigh, respectively (Figure 2). The administration of ceftibuten HSR resulted in bacterial reductions in all isolates (range: -0.49 to 1.43 log_10_ CFU/thigh), and five of the nine isolates achieved a 1-log_10_ CFU/thigh reduction. The composite exposure–response relationship for ceftibuten against individual isolates is depicted in Table 3. Reflecting the strain variability among these clinical isolates, the exposure–response relationships for the isolates were also variable, based on the coefficient of determination (average R^2^ = 0.79, range: 0.39 to 0.92); however, the majority were relatively robust (R^2^ > 0.70). Using these sigmoidal fits, three of the nine isolates did not achieve a 1-log_10_ CFU/thigh reduction, while one isolate did not achieve stasis. The *f*T > MICs corresponding to the stasis and 1-log_10_ reduction for each individual isolate are provided in Table 3. On average, the *f*T > MIC required for the bacteriostasis and 1-log_10_ reduction against the five *E. coli* isolates evaluated was 34% and 61%, respectively. A higher *f*T > MIC threshold for the stasis (44%) and 1-log_10_ reduction (77%) was observed against the four *K. pneumoniae* isolates evaluated. The aggregate static targets for wild-type isolates were similar to the ESBL-harboring isolates (*f*T > MIC 32% vs. 31%). Based on the composite exposure–response data from all isolates (Figure 3), the ceftibuten *f*T > MIC values associated with the static and 1-log_10_ targets for these nine isolates were 39% and 67%, respectively.

## 3. Discussion

The combination of novel BLIs with already-approved β-lactam agents continues to be a safe and attractive drug development strategy for combating the growing threat of antimicrobial resistance [6]. Although the majority of combination products in the pipeline are intravenous, a push towards the development of oral combination products to serve as a step-down option or to facilitate outpatient management is gaining momentum [7,16]. Characterizing the range of microbiological activity of β-lactam is imperative for understanding its potential activity against antibiotic resistant isolates when combined with a BLI. Therefore, this in vivo study sought to bridge the gap in ceftibuten pharmacodynamic knowledge and provide relevant exposure–response data against wild-type and ESBL-harboring isolates.

The ceftibuten dose-ranging experiments in this study demonstrated that a mean ceftibuten *f*T > MIC of 67% translated into a 1-log_10_ CFU/thigh reduction in bacterial burden. This data provide a ceftibuten-specific target, negating the extrapolation from ranges (40–70%) determined from other cephalosporin pharmacodynamic studies [10,11,12]. In addition, net stasis, an adopted microbiological surrogate for clinical efficacy in urinary tract infections (UTIs), [17] was observed at a ceftibuten *f*T > MIC of 39%.

Enzymatic mechanisms of resistance continue to be a major burden on public health, and ESBL-harboring isolates are no exception. ESBL-harboring Enterobaterales isolates continue to be a contributor to patient morbidity and mortality, with incidence increasing by 53% from 2012 to 2017 [18]. Unlike other forms of enzymatic resistance, ESBLs can commonly manifest in community and hospital-acquired infections [1]. The development of an appropriate oral BL/BLI against this infection entity is imperative. Ceftibuten is stable against narrow-spectrum ESBLs, but is readily hydrolyzed by broader spectrum variants [8,9]. In this study, the pharmacodynamic targets for ESBL-harboring isolates, albeit limited by the small sample size, were comparable to their wild-type counterpart, and were similar to previous findings [19]. In addition to classic dose-ranging studies, we also utilized a ceftibuten human-simulated regimen to evaluate the bactericidal activity of clinically-relevant ceftibuten exposure. Concordant with its clinical utility for UTIs, ceftibuten at an exposure equivalent to that of a human oral dose of 300 mg taken orally every 8 h resulted in bacterial stasis in all isolates and achieved a 1-log_10_ CFU/thigh reduction in five isolates. This regimen can be used in conjunction with a novel β-lactamase-inhibitor to optimize exposure profiles against ESBL- and carbapenemase-harboring isolates in vivo. Further studies evaluating the therapeutic potential of ceftibuten drug combinations are warranted.

## 4. Materials and Methods

### 4.1. Antimicrobial Test Agents

Analytical grade ceftibuten (lot RCHX170002, Covalent Laboratories Private Limited, Hyderabad, India) was reconstituted and diluted to the desired dosing concentrations in a 50 mM sodium phosphate buffer. All doses were administered subcutaneously with a final volume of 0.2 mL.

### 4.2. Isolates

A total of nine clinical Enterobacterales isolates were obtained from the Centers for Disease Control and Food and Drug Administration Antimicrobial Resistance Isolate Bank (*n* = 1), the Antibacterial Research Leadership Group isolate bank (*n* = 5), the American Type Culture Collection (*n* = 2), and the Center for Anti-Infective Research and Development (CAIRD) isolate library (*n*=1). *Escherichia coli* (*n* = 5) and *Klebsiella pneumoniae* (*n* = 4) isolates were selected for their ESBL-harboring and wild-type genotypes. The ceftibuten broth microdilution MICs ranged from 0.03–4 mg/L. The individual isolate genotype and ceftibuten MICs are available in Table 4. Notably, one *K. pneumoniae* isolate (KP 956) was characterized as SHV-harboring, but displayed the phenotypic profile (ceftibuten MIC: 0.03 mg/L) of a wild-type isolate and thus was included in the wild-type isolate analysis. All of the isolates were stored frozen at −80 °C in skim milk. Prior to examination, each isolate was sub-cultured twice on trypticase soy agar with 5% sheep blood (Becton Dickinson and Co., Sparks, MD, USA) and incubated at 37 °C for 18–24 h.

### 4.3. Animals

Specific-pathogen-free, female, CD-1 mice (20–22 g) were obtained from Charles River Laboratories, Inc. (Raleigh, NC, USA). All animals were allowed to acclimatize for 48 h prior to the study procedures, and were housed in groups of six animals at controlled room temperature in HEPA-filtered cages (Innovive, San Diego, CA, USA). The cages were supplemented with paper nesting material for enrichment purposes. Study rooms were maintained with diurnal cycles (12 h light/12 h dark), and food and water were provided ad libitum. Animals were monitored three times daily for signs of morbidity and were euthanized if found moribund; tissues were harvested subsequent to euthanasia.

### 4.4. Neutropenic Thigh Infection Model

Prior to both the pharmacokinetic and in vivo efficacy studies, the animals were prepared as follows: neutropenia was induced by administering 150 mg/kg of intraperitoneal (i.p.) cyclophosphamide on day 4 and 100 mg/kg on day 1. In addition, a predictable degree of renal impairment was produced using 5 mg/kg of uranyl nitrate administered i.p. on day 3 [20]. Bacterial suspensions of ~1 × 10^7^ colony forming units (CFU)/mL in normal saline were used for the inoculation of both thighs (injection volume 0.1 mL) 2 h prior to the first antibacterial dose.

### 4.5. Murine Pharmacokinetic Studies

The ceftibuten pharmacokinetic parameters were derived from single dose pharmacokinetic studies. Briefly, six mice per time point were prepared for experimentation as described above, and were then subjected to a single dose of ceftibuten (0.5 mg/kg, 1.5 mg/kg, 6 mg/kg, 20 mg/kg, and 45 mg/kg). The mice were euthanized by CO_2_-asphyxiation and blood samples were obtained by cardiac puncture. Six time-points were assessed (0.25 h, 0.5 h, 1 h, 2 h, 4 h, and 8 h). Blood was collected in K_2_EDTA Tubes (Becton Dickinson and Co., Sparks, MD, USA) and centrifuged at 10,000 rpm for 10 min at 8 °C. The separated plasma was stored at −80 °C until the total drug concentrations were determined using an ultra-performance liquid chromatography with tandem mass spectrometry method. The pharmacokinetic parameters were calculated using the mean drug concentrations from each group of mice, while the AUC was estimated using the linear-up log-down trapezoidal rule. Using the pharmacokinetic parameter estimates derived from the single dose pharmacokinetic studies, concentration–time profiles over 24 h were simulated to obtain *f*T > MIC values for the respective doses administered. All pharmacokinetic analyses were performed in Phoenix WinNonlin (Pharsight Corp., Mountainview, CA, USA). The final weighting schemes were decided on by considering the Akaike information criterion and best visual fit. The averaged pharmacokinetic parameters were also used to develop a human-simulated regimen. Simulated-free ceftibuten concentrations were determined by considering the extent of murine protein binding (19.7%) [13].

### 4.6. Pharmacodynamic Studies

Eight ceftibuten regimens with doses ranging from 0.5 to 45 mg/kg over a frequency of once to every 3 h were developed to achieve various *f*T > MICs. In addition, a previously developed ceftibuten human-simulated dosing regimen (HSR) was administered to achieve plasma exposures similar to those achieved in humans following an oral dose of 300 mg of ceftibuten every 8 h [15,21]. During experimentation, 7 groups (2 control and 5 treatment groups) of 3 mice each were inoculated with the respective isolates. Two hours after thigh inoculation, one group was sacrificed at 0 h via CO_2_-asphxyation and cervical dislocation in order to determine the baseline bacterial burden. The remaining 6 groups received a subcutaneous injection of one of the following regimens for 24 h: ceftibuten HSR, a selected ceftibuten regimen to achieve a targeted *f*T > MIC, or an injection of 0.9% normal saline (NS) given at the same frequency as the ceftibuten HSR. After 24 h, all treatment groups were euthanized and the thighs (*n* = 6/group) were aseptically harvested and homogenized in NS. Each thigh was treated as an independent value. The homogenized thigh was serially diluted onto trypticase soy agar with 5% sheep blood (Becton Dickinson and Co., Sparks, MD, USA), and colonies were enumerated to determine the number of CFU per thigh after incubation overnight. The efficacy of each regimen was determined using the change in log_10_ CFU/thigh from the 0 h control. Log_10_ change in CFU/thigh was reported as mean ± SD for ceftibuten HSR. A uniform E_max_ model using the Hill equation was fitted to the *f*T > MIC vs. change in bacterial burden at 24 h using Phoenix WinNonlin for individual isolates. An aggregate composite model derived from the averaged E_max_ model parameters of all bacterial strain data was constructed. Similarly, composite profiles for all the wild-type, ESBL-harboring, *E. coli*, and *K. pneumonia* isolates were also constructed using their respective strain data. These models were used to calculate effective stasis and 1-log_10_ reduction pharmacodynamic targets for individual isolates and the aggregate composite.

## 5. Conclusions

To the best of our knowledge, this is the first study examining the pharmacodynamics of ceftibuten. In conjunction with the classic exposure–response profiles, the clinical exposure assessment using the human-simulated regimen provides a framework for the selection of ceftibuten as a partner agent in BL/BLI oral combinations against isolates frequently causing urinary tract infections. Additional studies evaluating these types of drug combinations are warranted.

## Figures and Tables

**Table 1:** Comparison of single dose ceftibuten regimens in mice using non-compartmental analysis.

Single Dose Regimen	λ_1/2_ (h)	*f*Cmax	*f*AUC_0–8_	R^2^
0.5 mg/kg	1.0	1.0	2.3	0.90
1.5 mg/kg	1.2	5.1	6.8	0.92
6 mg/kg	1.5	12.8	37.0	0.98
20 mg/kg	1.8	40.4	119.3	0.99
45 mg/kg	1.4	95.6	191.0	0.97

λ_1/2_—lambda half-life; *f*Cmax—free maximum concentration; *f*AUC_0–8_—free area under the curve from 0–8 h.

**Table 2:** Comparison of the %*f*T > MIC values achieved with ceftibuten regimens at different MICs in humans and the murine thigh infection model.

Dosing Regimen	%*f*T > MIC (mg/L)								*f*AUC_0–24_	C_max_
	0.03	0.06	0.125	0.25	0.5	1	2	4		
CTB 300 mg q8 h (human) [15]	100%	100%	100%	100%	100%	91%	59%	0%	56.2	3.75
CTB 300 mg q8 h HSR (murine)	100%	100%	100%	100%	100%	91%	50%	0%	50.1	3.6
0.5 mg/kg single dose	24%	22%	17%	12%	7%	0%	0%	0%	2.1	0.97
3 mg/kg single dose	37%	35%	30%	25%	20%	15%	10%	4%	12.8	5.8
0.5 mg/kg q8 h	71%	66%	51%	36%	21%	0%	0%	0%	6.4	0.98
1 mg/kg q6 h	100%	100%	90%	70%	48%	28%	1%	0%	16.89	2.02
6 mg/kg q8 h	100%	100%	100%	91%	76%	61%	45%	30%	76.4	11.8
20 mg/kg q6 h	100%	100%	100%	100%	100%	100%	96%	76%	337.8	40.3
20 mg/kg q4 h	100%	100%	100%	100%	100%	100%	100%	100%	500.7	43.8
45 mg/kg q3 h	100%	100%	100%	100%	100%	100%	100%	100%	1487.6	108.7

*f*T > MIC—free time above the MIC; *f*AUC_0–24_—free area under the curve for a 24-h period; C_max_—maximum concentration; HSR—human-simulated regimen.

**Table 3:** %*f*T > MIC pharmacodynamic targets for ceftibuten against individual isolates and the aggregate composite.

Isolate (MIC (mg/L))	Ceftibuten %*f*T > MIC Required to Achieve		R^2^
	Stasis	1-log Reduction	
KP 956 (0.03)	46	68	0.90
EC 756 (0.125)	17	35	0.72
KP 957 (0.125)	NA	NA	0.74
EC 551 (0.25)	35	NA	0.85
KP 958 (0.25)	40	59	0.92
EC 25922 (0.5)	26	56	0.78
EC 761 (1)	17	31	0.9
KP 700603 (1)	44	NA	0.89
EC 643 (4)	11	69	0.39
Median ^1^	31	58	-
IQR ^1^	17–41	40–66	0.74–0.9

^1^—calculated from isolates that achieved the target goal; NA—not achieved.

**Table 4:** Genotypic and phenotypic profiles of ceftibuten against the test isolates.

Organism	CAIRD ID	Strain	Known Resistance Mechanism(s)	Ceftibuten MIC (mg/L)
*Klebsiella pneumoniae*	956	ARLG 1112	SHV	0.03
*Escherichia coli*	756	ARLG 1023	None	0.12
*Klebsiella pneumoniae*	957	ARLG 1118	None	0.12
*Escherichia coli*	551	CDC 0077	None	0.25
*Klebsiella pneumoniae*	958	ARLG 1120	None	0.25
*Escherichia coli*	25922	ATCC 25922	None	0.5
*Escherichia coli*	761	ARLG 1050	None	1
*Klebsiella pneumoniae*	700603 *	ATCC 700603	SHV-18, OXA-2, OKP-B-6	1
*Escherichia coli*	643 *	SI-LP377	CTX-M2	4

CAIRD—Center for Anti-Infective Research and Development; ARLG—Antibacterial Research Leadership Group; CDC—Centers for Disease Control; ATCC—American Type Culture Collection. * ESBL-harboring isolates.

## Data Availability

Data is available upon reasonable request to corresponding author.
